# Diagnostic Performance of a Magnetic Field-Enhanced Agglutination Readout in Detecting Either Viral Genomes or Host Antibodies in Arbovirus Infection

**DOI:** 10.3390/microorganisms9040674

**Published:** 2021-03-24

**Authors:** Fanny Leon, Elena Pinchon, Nevzat Temurok, François Morvan, Jean-Jacques Vasseur, Martine Clot, Vincent Foulongne, Jean-François Cantaloube, Philippe Vande Perre, Jean-Pierre Molès, Aurélien Daynès, Chantal Fournier-Wirth

**Affiliations:** 1 Pathogénèse et Contrôle des Infections Chroniques et Emergentes, Université de Montpellier, Etablissement Français du Sang, Inserm, Université des Antilles, 34184 Montpellier, France; fanny.leon@efs.sante.fr (F.L.); elena.pinchon@efs.sante.fr (E.P.); v-foulongne@chu-montpellier.fr (V.F.); jean-francois.cantaloube@efs.sante.fr (J.-F.C.); p-van_de_perre@chu-montpellier.fr (P.V.P.); jean-pierre.moles@inserm.fr (J.-P.M.); 2 HORIBA Medical, 34184 Montpellier, France; nevzat.temurok@horiba.com (N.T.); martine.clot@horiba.com (M.C.); aurelien.daynes@horiba.com (A.D.); 3 Institut des Biomolecules Max Mousseron (IBMM), Université de Montpellier, CNRS, ENSCM, 34095 Montpellier, France; francois.morvan@umontpellier.fr (F.M.); jean-jacques.vasseur@umontpellier.fr (J.-J.V.)

**Keywords:** arbovirus, innovative diagnostic, magnetic agglutination, nanoparticles, viral genomes, antibodies

## Abstract

Arbovirus diagnostics on blood from donors and travelers returning from endemic areas is increasingly important for better patient management and epidemiological surveillance. We developed a flexible approach based on a magnetic field-enhanced agglutination (MFEA) readout to detect either genomes or host-derived antibodies. Dengue viruses (DENVs) were selected as models. For genome detection, a pan-flavivirus amplification was performed before capture of biotinylated amplicons between magnetic nanoparticles (MNPs) grafted with DENV probes and anti-biotin antibodies. Magnetization cycles accelerated this chaining process to within 5 min while simple turbidimetry measured the signal. This molecular MFEA readout was evaluated on 43 DENV RNA(+) and 32 DENV RNA(−) samples previously screened by real-time RT-PCR. The sensitivity and the specificity were 88.37% (95% CI, 78.76%–97.95%) and 96.87% (95% CI, 90.84%–100%), respectively. For anti-DENV antibody detection, 103 plasma samples from donors were first screened using ELISA assays. An immunological MFEA readout was then performed by adding MNPs grafted with viral antigens to the samples. Anti-DENV antibodies were detected with a sensitivity and specificity of 90.62% (95% CI, 83.50%–97.76%) and 97.44% (95% CI, 92.48%–100%), respectively. This adaptable approach offers flexibility to platforms dedicated to the screening of emerging infections.

## 1. Introduction

The emergence and re-emergence of arthropod-borne viruses (arboviruses) belonging to the *Flaviviridae*, *Togaviridae*, *Reoviridae*, or *Bunyaviridae* families constitute a threat to global health following their spread throughout all continents [1,2,3]. Dengue, selected as a model in this study, is the most prevalent arbovirus disease affecting humans with an estimated 390 million infections per year, 96 million of which are symptomatic, comprising 2 million severe with 21,000 fatal cases [4,5]. Dengue viruses (DENVs) belong to the genus *Flavivirus* that includes Zika, yellow fever, and West Nile viruses, and are transmitted to humans bitten by infected *Aedes aegypti* and *Aedes albopictus* mosquitoes [2,6]. Dengue disease is caused by four distinct viruses classified into serotypes 1–4. Infection with one serotype might confer lifelong immunity to that serotype but cross-immunity to the other serotypes after recovery is only partial. Areas at risk can be hyperendemic with co-circulation of multiple serotypes [4]. In its febrile phase, dengue cannot be clinically differentiated from other arboviruses or parasitic diseases due to common signs and symptoms in the infected host. 

The detection of DENV RNA in serum or plasma during the acute phase of infection provides a definitive and specific diagnosis of active infections [4,7]. This early diagnosis allows an improvement of the clinical management of severe dengue diseases and thus a reduction in mortality, while avoiding inappropriate treatment of patients with similar symptoms but no DENV infection [8]. Such molecular testing is only possible within the first week post-infection as DENV viremia is detectable 24–48 h before fever onset and continues for 5–6 days [4]. The quantitative real-time RT-qPCR method, combining reverse transcription and amplification of RNA genomes with a fluorescence detection step on sophisticated thermal cyclers, is the standard molecular method used in the clinical diagnosis of DENV infections with a high level of sensitivity and specificity. A simplified method of diagnosis during the acute phase of infection consists of the detection of DENV NS1 antigen, but this approach remains less sensitive compared to RNA detection [4,7,9,10]. The development of multiplex RT-qPCR assays is important to discriminate co-circulating arboviruses in endemic areas [9]. We previously developed a multiplex approach based on a pan-flavivirus RT-PCR amplification combined with specific capture of amplified genomes on tetrathiolated probes grafted on microplates [11]. In its early stage of development, molecular hybridization events were detected by time-resolved fluorescence using a microplate reader.

In order to develop a fast and low-cost DNA detection approach avoiding fluorescence readout, we more recently carried out a proof-of-concept study using synthetic DNA sequences or cultivated viruses to describe a simple detection readout by magnetic field-enhanced agglutination (MFEA) [12]. 

Seroconversion of anti-DENV IgM or IgG antibodies is the standard diagnostic method for serologically confirming a DENV infection [4]. Furthermore, one vaccine, Dengvaxia, is available for patients aged 9–45 years only and with confirmed past DENV infections but not dengue-naïve individuals (https://www.cdc.gov/dengue/prevention/dengue-vaccine.html, accessed on 15 February 2021). In this context, the diagnosis of anti-DENV host responses is important not only to diagnose convalescent patients during the chronic phase of infection but also prior to vaccination for selecting eligible individuals. Serological assays are mostly based on highly sensitive enzyme-linked immunosorbent assay (ELISA) and rapid immunochromographic strips [4,7]. In order to combine sensitivity and speed in obtaining results, we have worked on a rapid serological assay using magnetic field-enhanced and agglutination to detect host antibodies (unpublished data). The major challenge of DENV diagnosis by serologic analysis is the extensive cross-reactivity of antibody responses resulting from prior flaviviral infections and/or vaccination. Serological assay alone cannot discriminate primary from secondary DENV infections [9,13]. The current diagnosis of dengue and other flavivirus-associated human diseases requires laboratory testing and different sophisticated instruments and platforms for the direct detection of the infecting agent during the acute phase of infection or the detection of antibodies during the convalescent phase [10,13].

In the present work, magnetic nanoparticles (MNPs) were grafted either with tetrathiolated DNA probes, to detect specifically amplified DENV genomes, or with DENV NS1 antigens to detect host antibodies. The diagnostic performance, sensitivity, specificity, and accuracy of the molecular and immunological DENV MFEA approaches were determined in biological samples from patients or healthy blood donors. Our results are encouraging for the further development of this simple, very fast, and flexible MFEA readout for arbovirus diagnostics in general.

## 2. Materials and Methods

### 2.1. Biological Samples

To develop the molecular MFEA readout, plasma samples from 32 blood donors with no history of contact with arboviruses (DENV(−) RNA samples), collected by the French blood establishment (Etablissement Français du Sang; EFS) in Montpellier, Metropolitan France, were used as negative controls. A panel of 43 DENV(+) RNA samples extracted from human plasma were provided as frozen vials by the French national arbovirus surveillance center (Centre National de Référence des arbovirus; CNR) in Marseille, France. DENV RNA levels were measured before shipment by the CNR using a reverse transcription (RT)-quantitative real-time PCR (qPCR) method (RT-qPCR). A cycle threshold (Ct), defined as the number of cycles required for the fluorescent signal to exceed the background level, of over 30 was indicative of a very low viral load (Supplemental Table S1). Due to de-identification of the participants, no demographics were recorded and only DENV serotype and Ct value were used in this study. 

Cross-reactivity was evaluated on eight replicates using samples tested positive for the molecular presence of Zika (ZIKV, 10^4^ TCID_50_/mL), Chikungunya (CHIKV, 10^6^ TCID_50_/mL), West Nile virus (WNV, 15 copies/mL), human immunodeficiency virus (HIV, 29,532 copies/mL), or hepatitis C virus (HCV 534,250 UI/mL) (Supplemental Figure S1).

To develop the immunological MFEA readout, 103 blood donor plasma samples collected by the EFS in endemic areas (French West Indies) and one non-endemic area (Montpellier) were screened for the presence of DENV IgM or IgG antibodies using two commercial ELISAs (InBios International, Inc., Seattle, WA, USA), the qualitative DENV Detect IgM Capture ELISA Kit (FDA cleared), and the DENV Detect IgG ELISA kit (CE approved), respectively. These characterized samples were aliquoted and stored at −80 °C until use.

### 2.2. Viral Nucleic Acid Extraction and Amplification

Viral nucleic acid extraction was performed using the MagNA Pure Compact automated system with the MagNA Pure Compact Nucleic Acid Isolation Kit according to the manufacturer’s instructions (Roche Diagnostics, Mannheim, Germany). An input volume of 200 µL of plasma from blood donors and an elution volume of 50 µL were chosen. The purified viral nucleic acids were aliquoted and stored at −80 °C until their use. A pan-flavivirus conventional one step RT-PCR amplification (Qiagen, Valencia, CA, USA) targeting the flavivirus NS5 gene and avoiding the use of real-time RT-qPCR and fluorescent probes was used. The MAMD forward primer (5′AAC ATG GGR AAR AGR GAR AA3′) was 5′-tagged with biotin to generate biotinylated amplified genomes. Asymmetric RT-PCR amplification was carried out using 5 µL of extracted viral RNAs mixed with 3 µL of MAMD forward primer (10 µM) and 0.3 µL of cFD2 reverse primer (5′GTG TCC CAG CCG GCG GTG TCA GC3′; 10 µM) in a final volume of 50 µL [12,14]. The total amplification time was 2.5 h [12]. The PCR procedures were performed using a T Advanced Biometra thermal cycler (Analytik Jena AG, Germany) and amplified DENV DNAs were tested immediately or stored at −20 °C until their use.

### 2.3. Grafting of Tetrathiolated DENV Probes onto Magnetic Nanoparticles

The 5’-tetrathiolated DENV probe 5′TCC TTC YAC TCC RCT3′ was synthesized on a 1 µmol scale using a DNA synthesizer, and lyophilized before use [11,12]. This DENV probe, designed to detect the four serotypes of DENV genomes, was covalently grafted onto 200 nm diameter MNPs (200 nm carboxyl-adembeads, Ademtech, Pessac France) using an amino-polyethylene glycol (PEG)-maleimide crosslinker as previously described [12]. The MNPs were passivated by incubations with 1 mL of 1.5 M Tris-HCl, pH 8.8, for 20 min and 250 µL of a cysteine solution (80 mg/mL) for 10 min. The MNPs covalently grafted with the DENV probe (MNPs-Probe) were stored at 1% *w*/*v* in a dedicated buffer (10 mM glycine, 0.02% NaN_3_, 0.1% Synperonic F108 non-ionic surfactant, pH 9) for up to 6 months at 4 °C.

### 2.4. Grafting of DENV NS1 Antigens onto Magnetic Nanoparticles

MNPs (200 nm carboxyl-adembeads, Ademtech, Pessac France) were first activated with EDC (1-ethyl-3-(3-dimethyaminopropyl) carbodiimide hydrochloride) to form an amine-reactive intermediate, as recommended by the manufacturer. The activated MNPs were then coated with 20 µg of DENV NS1 antigen (PIP047B Biorad, Marnes-la-Coquette, France) per mg of MNPs for 2 h at 37 °C under shaking. The MNPs were then washed twice and suspended at 1% *w/v* in a 10 mM glycine buffer at pH 9, with 0.1% Synperonic F108 non-ionic surfactant (Sigma Aldrich, Saint Quentin Fallavier, France) and 0.02% sodium azide as a preservative. The MNPs grafted with DENV NS1 antigen (MNPs-NS1) were stored for up to 6 months at 4 °C until use.

### 2.5. Molecular MFEA Readout

The molecular detection step was performed in a disposable spectrophotometric cuvette surrounded by an electromagnet that provided a 15 mT magnetic field. This prototype included a LED source emitting at 650 nm and a photodiode to perform a simple optical detection [12].

**Figure 1:**
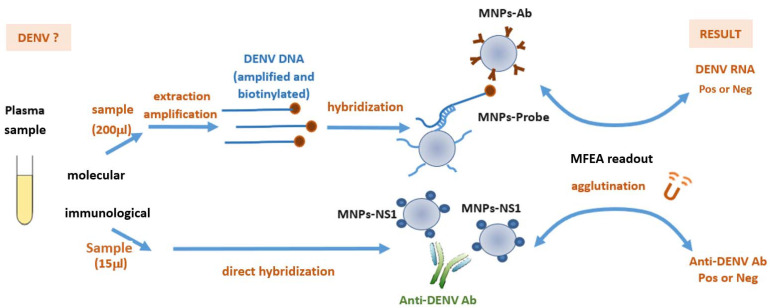
Diagnostic of dengue virus (DENV) infection by magnetic field-enhanced agglutination readout (MFEA readout). The molecular MFEA readout aims to detect the DENV RNA during the acute phase of infection. DENV genomes are extracted and amplified using asymmetric pan-flavivirus RT-PCR amplification. The biotinylated DENV amplicons are captured between magnetic nanoparticles (MNPs) grafted with specific DENV tetrathiolated DNA probes (MNPs-Probe) and anti-biotin antibodies (MNPs-Ab). The immunological MFEA approach aims to detect the anti-DENV antibodies in plasma samples during the convalescent phase of infection. These antibodies are captured between MNPs grafted with viral DENV NS1 antigens (MNPs-NS1) in a homogeneous phase.

The amplified and biotinylated single-stranded DENV DNAs were detected by MFEA readout using MNPs onto which either DENV probes (MNPs-Probe) or anti-biotin antibodies (MNPs-Ab) had been grafted (Figure 1). The MNPs-Ab were prepared using a carbodiimide coupling chemistry by adding 20 µg of anti-biotin antibody (Jackson ImmunoResearch Europe LTD, Cambridge, UK) to 1 mg of MNPs as previously described [12]. Amplified DENV DNAs were diluted 1:10 in hybridization buffer (HB) (6X SSPE, 5X Denhardt solution) before incubation with MNPs-Probe for 5 min at 37 °C under agitation. This mix was then transferred into two disposable cuvettes containing MNPs-Ab to perform the agglutination assay by applying three cycles of magnetization (60 s) and relaxation (30 s) [12]. The turbidity signal was expressed as the total variation of optical density at 650 nm (ΔOD_650nm_) measured before and after the three magnetization cycles. The total detection time was less than 5 min. All measurements were performed in duplicate. Synthetic 15-mer DENV DNA oligonucleotides biotinylated at their 5′-end were used at 1000 pM as positive controls in each assay. Samples containing HB, MNPs-Probe, and MNPs-Ab, and RT-PCR mix without DENV genome were defined as blank samples (devoid of analyte).

### 2.6. Immunological MFEA Readout

Plasma samples (15 µL) were added to a disposable cuvette containing 57 µL of reaction buffer (RB) (50 mM HEPES buffer, pH 7.5, 0.8% Synperonic F108, 800 mM NaCl, and 0.09% sodium azide). MNPs-NS1 were then added at a final concentration of 0.04% *w*/*v* (Figure 1). The detection of anti-DENV antibodies was performed directly in a homogeneous phase. The cuvette was inserted into the prototype and a 15 mT magnetic field then applied to accelerate the capture of anti-DENV antibodies by the MNPs-NS1. One cycle of magnetization (60 s) and relaxation (30 s) led to the progressive formation of aggregates. The turbidity signal was expressed as the total variation of optical density at 650 nm (ΔOD_650nm_) measured before and after the magnetization cycle. The total detection time was less than 5 min.

### 2.7. Statistical Analysis

The data in Tables 1 and 2 represent the results obtained for each biological sample tested in duplicate. The 95% confidence intervals (CI) for a proportion were calculated according to the method described by Robert Newcombe [15].

We used GraphPad Prism 8.0 software for generating scatterplots. To report the performance of the immunological MFEA readout, a receiver operating characteristic (ROC) curve was created using GraphPad Prism 8.0 software. The ROC curve presents test performance as true-positive (% sensitivity) versus false-positive fraction (100 − specificity %). The optimal cut-off value, which maximizes sensitivity and specificity, was calculated from the ROC curve.

## 3. Results

### 3.1. Sample Characteristics

A panel of 43 DENV(+) RNA samples from patients with different DENV serotypes was used in this study (Supplemental Table S1). A total of 32 plasma samples from blood donors collected in a non-endemic area were used as negative controls. All these samples were amplified using a pan-flavivirus conventional one step RT-PCR method [11] and detected by gel electrophoresis to control the biological material before its use. DENV amplified genomes were detected in 95% of DENV1 samples (*n* = 19/20), 90% of DENV2 samples (*n* = 9/10), 100% of DENV3 samples (*n* = 7/7), and 83.33% of DENV4 samples (*n* = 5/6) (Supplemental Table S1). No false-positive signals were detected on the agarose gel in the negative plasma controls. All 75 samples were included for molecular MFEA analysis. For the development of the immunological readout, 103 plasma samples from blood donors collected in endemic and non- endemic areas were tested using commercial ELISA assays for the presence of anti-DENV antibodies. Among them, 64 plasma samples were classified as positive for the presence of anti-DENV antibody (IgM(+): *n* = 1; IgM(+) and IgG(+): *n* = 3; IgG(+): *n* = 60; IgM(+) or IgG(+): *n* = 64). No signal was observed for 39 plasma samples classified as negative samples. All these 103 samples were used to test the performance of the immunological MFEA readout.

### 3.2. Diagnostic Performance of the Molecular MFEA Readout

**Figure 2:**
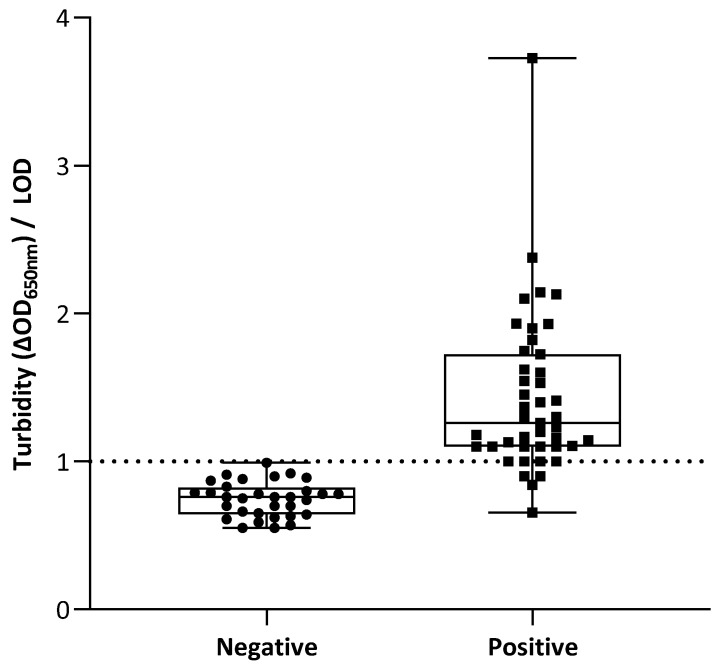
Molecular MFEA readout for DENV RNA(−) and DENV RNA(+) plasma samples.

We previously observed a correlation between viral loads (expressed in TCID_50_/mL using titrated supernatants from cell cultures infected with DENV) and turbidity signals indicating semi-quantitative measurements [12]. The discrimination potential of the molecular MFEA readout on human plasma samples is displayed in Figure 2. No correlation between viral loads (expressed in Ct values) and turbidity signals was obtained after one, two, or three cycles of magnetization (data not shown). We therefore established the limit of detection (LOD) by determining the mean value of blank samples plus three times the standard deviation, and data were used qualitatively [16]. Discrepancy in the results obtained on four positive samples using our approach and those obtained with the reference real-time RT-qPCR method corresponded to DENV samples with very low viral loads (Ct values > 30 (Supplemental Table S1). One positive out of six DENV4 samples using the reference method was not detectable by the MFEA assay. This sample, presenting a high viral load, is however detectable by electrophoresis after pan-flavivirus amplification, suggesting the presence of potential mutations and a lack of capture of this particular DENV4 genome onto the MNPs-Probe. This hypothesis was, however, not confirmed due to insufficient volume disallowing any sequencing. Overall, our molecular MFEA readout detected 38 out of 43 DENV positive cases (88.37% diagnostic sensitivity; 95% CI, 78.79%–97.95%) (Table 1). The ability to correctly differentiate DENV-infected and negative samples is indicated by the accuracy value of 92%. All but one negative plasma samples (*n* = 31/32) were correctly identified as healthy (96.87% diagnostic specificity; 95% CI, 90.84%–100%). No cross-reaction was observed when testing other viruses (ZIKV, CHKV, WNV, HIV and HCV) with the pan-flavivirus RT-PCR amplification and the MFEA detection (Supplemental Figure S1).

Negative plasma from donors (*n* = 32) and positive plasma samples from patients (*n* = 43) were assayed. The turbidity signal is expressed as the difference of optical density at 650 nm (ΔOD_650nm_) measured before and after the three magnetization cycles. The limit of detection (LOD) is taken as the mean value of blank samples plus three standard deviations. Individual points of the scatterplot represent the ratio of turbidity signal/LOD calculated for one sample by the molecular MFEA readout. Data are expressed as median ratios with interquartile ranges.

**Table 1:** Molecular MFEA readout on biological samples.

Sample Type	Samples, *n*	Samples Correctly Detected, *n*	Diagnostic Sensitivity * % (95% CI)	Diagnostic Specificity ^†^ % (95% CI)	Accuracy ^‡^ %
**DENV**	43	38	88.37 (78.79–97.95)	/	92
**Healthy**	32	31	/	96.87 (90.84–100.00)	

Detailed results can be found in Supplemental Table S1. DENV, dengue virus; CI, confidence interval. * [number of positive samples/(number of positive samples + number of false-negative samples)] × 100. ^†^ [number of negative samples/(number of negative samples + number of false-positive samples)] × 100. ^‡^ [(number of negative samples + number of positive samples)/(number of negative samples + number of positive samples + number of false-negative samples + number of false-positive samples)] × 100.

### 3.3. Diagnostic Performance of the Immunological MFEA Readout

**Figure 3:**
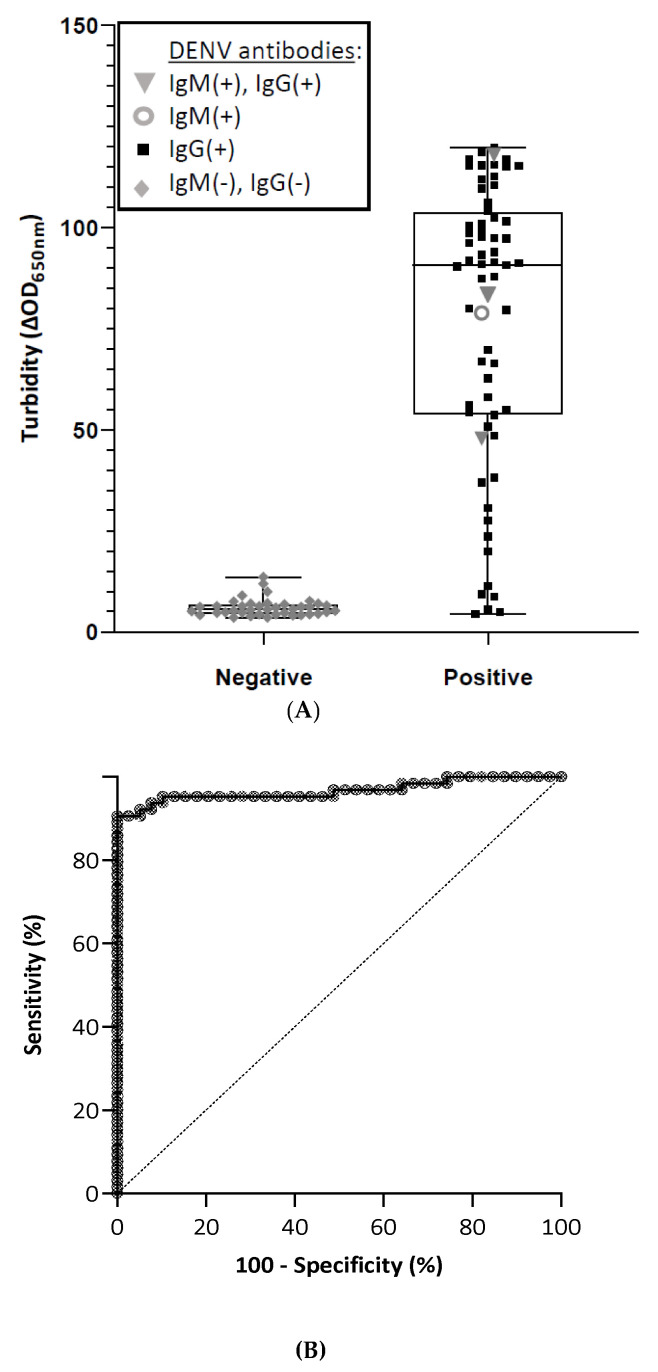
Performance of the immunological MFEA readout at detecting anti-DENV antibodies. (**A**) Scatterplots of the turbidity signal obtained in plasma samples classified as negative or positive for the presence of anti-DENV antibodies. Data are expressed as median turbidity signals with interquartile ranges. (**B**) Receiver operating characteristics (ROC) curve. The plot of True-Positive Fraction (sensitivity %) (true-positive samples/true-positive plus false-negative samples) vs. False-Positive Fraction (100 − specificity %) (false-positive samples/false-positive plus true-negative samples) generates the ROC curve. AUC: area under the ROC curve.

DENV virions and NS1 antigen circulate throughout the acute phase of infection. The DENV NS1 antigen can be endoplasmic reticulum-anchored, membrane-associated or secreted to activate the innate immune system [4]. This viral antigen was selected as a biorecognition element to be grafted onto MNPs in order to detect the anti-NS1 antibodies potentially present in the tested plasma sample. The immunological MFEA approach was performed on 39 negative and 64 positive plasma samples as previously described. The test was performed in all plasma samples after a 1:5 dilution in reaction medium. As shown in Figure 3, the median turbidity was significantly lower for negative plasma samples vs. those that were classified as positive (5.55 [4.50–6.83] vs. 90.75 [53.8–103.7] respectively). The receiver operating characteristic (ROC) curve analysis demonstrated very good discrimination between negative and convalescent DENV plasma samples, with an area under the ROC curve of 0.967 (95% CI, 0.935–1.00). The optimal cut-off value for turbidity was 12.74 mOD. This value was then used to study the diagnostic performance of the immunological readout (Table 2). Our immunological MFEA readout detected 58 out of 64 DENV positive cases (90.62% diagnostic sensitivity; 95% CI, 83.50%–97.76%) (Table 2) with 93.20% accuracy. All but one negative sample (*n* = 38/39) were correctly tested negative for the presence of anti-DENV antibodies (97.44% diagnostic specificity; 95% CI, 92.48%–100%) (Table 2).

**Table 2:** Immunological MFEA readout in biological samples.

Sample Type	Samples, *n*	Samples Correctly Detected, *n*	Diagnostic Sensitivity * % (95% CI)	Diagnostic Specificity ^†^ % (95% CI)	Accuracy ^‡^ %
**DENV**	64	58	90.62 (83.50–97.76)	/	93.20
**Healthy**	39	38	/	97.44 (92.48–100.00)	

DENV, dengue virus; CI, confidence interval. * [number of positive samples/(number of positive samples + number of false-negative samples)] × 100. ^†^ [number of negative samples/(number of negative samples + number of false-positive samples)] × 100. ^‡^ [(number of negative samples + number of positive samples)/(number of negative samples + number of positive samples + number of false-negative samples + number of false-positive samples)] × 100.

## 4. Discussion

Over the last few decades, arboviruses have considerably expanded their geographic range and their impact on public health is worsening [1,2,17]. The epidemic of arbovirus diseases observed over the past 50 years has arisen due to several factors increasing the risk of human exposure, including land use and urbanization, high population density, globalization of travel and trade, climate change, and the expansion of vector habitats [17,18]. The burden of dengue is growing, and more than half of the world’s population (3.6 billion people) live in areas at risk of dengue transmission [4,5]. Moreover, a previous DENV infection increases the risk of severe infection with another DENV serotype as preexisting, non-neutralizing antibodies facilitate infection of Fcγ receptor-bearing cells [2,4,19,20]. DENV infects different organs and replicates in multiple cells [21]. There remain no specific antiviral therapies to treat dengue. The diagnosis of infectious diseases in at-risk areas is complex, and formalized programs and models are needed to help physicians improve patient care [22,23].

Multiple effective molecular technologies exist but mostly require expensive equipment and trained personnel. These limitations diminish their utility, in particular within resource-limited countries. In this context, the transition toward point-of-care systems has been highlighted as a priority for improving testing of infectious diseases [24]. Magnetic particles have been included in numerous optical or electrochemical biosensing technologies for lowering detection limits and nonspecific effects [25,26,27,28,29,30].

In this study performed on human plasma samples, we developed a new approach using magnetic nanoparticles grafted either with nucleic probe, to detect DENV RNA during the acute phase of infection, or with DENV NS1 antigen to detect the host antibodies. The instrument used to apply the magnetization cycles is very simple, consisting of a photodiode, an electromagnet, and a cuvette holder. The optical detection by turbidimetry avoids the use of costly fluorescence detection. The molecular MFEA approach combines a generic pan-flavivirus one step RT-PCR amplification on a simple thermocycler with a rapid optical detection based on a magnetic field-enhanced agglutination. The hybridization of amplicons on tetrathiolated probes improves the sensitivity and the specificity of detection. The molecular MFEA readout is able to detect DENV genomes with a diagnostic sensitivity of 88.37%. Only one false-positive signal was observed among the tested negative plasma samples and a diagnostic specificity of 96.87% was achieved. At this stage, our current data show that MFEA is not quantitative and that four out of five samples with high Ct values detected by RT-qPCR (Ct > 30) are not detected by MFEA. However, the RT-qPCR requires an instrument that operates in a lab environment. The MFEA, avoiding laser, may be used in other environments. The sensitivity and specificity provided in this study clearly show the good diagnostic performance of this strategy. Furthermore, with the emergence of new isothermal amplification methods (LAMP, RPA, RCA, etc.), innovative readouts could be very useful. Regarding the cost-effectiveness, the cost of TaqMan probes are not negligible, and a rapid cost analysis of our molecular assay will put the assay at one dollar per point. A generic DENV tetrathiolated probe aiming to detect the four DENV serotypes was designed in this study. This readout can be easily adapted for DENV genotyping using four type-specific DENV tetrathiolated probes if needed. In addition, as the genomes are amplified with a pan-flavivirus RT-PCR, the MFEA readout can readily be used for detecting other flavivirus, such as ZIKV, YFV, and WNV, by grafting dedicated tetrathiolated probes specific to each virus. The flexibility of detection is high for targeting different viruses using different magnetic particles. In order to simplify the pre-analytical phase, we plan to combine a rapid isothermal amplification method with the molecular MFEA readout in order to develop an automated and easy-to use platform.

This MFEA approach was then adapted to allow direct detection in a homogeneous phase, with no washing or incubation steps, of the presence of anti-DENV antibodies. The diagnostic sensitivity and the diagnostic specificity of the immunological MFEA readout was 90.62% and 97.44%, respectively. These findings are encouraging for the further development of the MFEA readout approach for the faster detection, in less than 5 min, of the presence of antibodies in a plasma sample by comparison with the reference ELISA methods requiring over 2 h. ELISA assays are usually not available in countries with limited access to laboratory facilities [13]. This immunological MFEA readout could represent an alternative approach for simple serological diagnosis. The main limitation of flavivirus serological assays is antibody cross-reactivity, that has been reported between flaviviruses members but also between flaviviruses and unrelated viruses such as alphaviruses or betacoronaviruses [13]. Furthermore, if subjects have been vaccinated with a flavivirus-based vaccine, the risk of cross-reactions with host neutralizing antibodies is possible and could alter the analytical performances of our approach. The possibility to functionalize MNPs using several DENV NS1 antigens with different epitopes recognized by host anti-DENV antibodies, or to use a cocktail of different MNPs each grafted with a specific DENV antigen, will open the way to improve the specificity of detection. Further clinical studies in endemic areas, with co-circulation of arboviruses, and in vaccinated populations will be needed to study cross-reactions on human samples. The immunological MFEA approach, combining an easy sample preparation and a very fast detection step, could be adapted to develop a simple sample-in-answer-out diagnostic system that requires minimal infrastructure to operate with high analytical performance.

Here, we have demonstrated the flexibility of the MFEA approach by its capacity to allow the use of nucleic probes or proteins as biorecognition elements for grafting onto MNPs that capture a dedicated target. This analytical molecular or immunological MFEA approach is potentially adaptable for use in point-of-care diagnostic systems or in high-throughput platforms. In the context of emergent arbovirus infections, the development of sensitive, specific, affordable, and rapid diagnostic systems or platforms is much needed to improve surveillance and diagnosis particularly in endemic countries.

## Data Availability

Not applicable.

## Supplementary Materials

The following are available online at https://www.mdpi.com/2076-2607/9/4/674/s1, Table S1: Full dataset of samples used in the molecular MFEA readout. Supplemental; Figure S1: Cross-reactivity of the molecular MFEA readout.
