# Supplementary material for: Diagnostic Performance of a Magnetic Field-Enhanced Agglutination Readout in Detecting Either Viral Genomes or Host Antibodies in Arbovirus Infection
Source: Microorganisms. 2021 Mar 24;9(4):674. doi: 10.3390/microorganisms9040674 (PMC8064388; doi:10.3390/microorganisms9040674)

Supplemental Table S1 Full data set of samples used in the molecular MFEA readout

Serotype	Sample	French national arbovirus surveillance center Real Time RT PCR (Ct value)	Electrophoresis gel	MNP Agglutination Turbidity
DENV1	1	8	+	+
	2	9	+	+
	3	9	+	+
	4	11	+	+
	5	11	+	+
	6	12	+	+
	7	13	+	+
	8	13	+	+
	9	13	+	+
	10	14	+	+
	11	18	+	+
	12	19	+	+
	13	19	+	+
	14	25	+	+
	15	27	+	+
	16	28	+/-	+
	17	28	+	+
	18	29	+	+
	19	31	+/-	-
	20	33	-	-

Serotype	Sample	French national arbovirus surveillance center Real Time RT PCR (Ct value)	Electrophoresis gel	MNP Agglutination Turbidity
DENV2	21	10	+	+
	22	12	+	+
	23	14	+	+
	24	14	+	+
	25	16	+	+
	26	16	+	+
	27	27	+	+
	28	30	+	+
	29	31	+/-	+
	30	32	-	-
DENV3	31	17	+	+
	32	18	+	+
	33	19	+	+
	34	23	+	+
	35	25	+	+
	36	26	+	+
	37	33	+	-
DENV4	38	10	+	+
	39	11	+	-
	40	13	+	+
	41	14	+	+
	42	19	+	+
	43	27	-	+
Total		43	37	38

Supplemental Figure S1 Cross reactivity of the molecular MFEA readout.
The turbidity signal is expressed as the difference of optical density at 650 nm (D OD_{650nm}) measured before and after the three magnetization cycles. The limit of detection (LOD) is taken as the mean value
of blank samples plus three standard deviations. The ratio of turbidity signal/LOD calculated by the molecular MFEA readout is represented for samples tested after a pan flavivirus amplification and a detection step using nanoparticles grafted with DENV probes. Cross reactivity was evaluated on eight replicates using samples tested positive for the molecular presence of the Zika (ZIKV), Chikungunya (CHIKV), West Nile (WNV), Human Immunodeficiency (HIV) or hepatitis C (HCV) viruses. A negative sample is represented by a ratio of turbidity signal/LOD under 1 (dashed line).
Synthetic 15-mer DENV DNA oligonucleotides (S1000) biotinylated at their 5'-end were used at 1000 pM as positive controls in each assay.

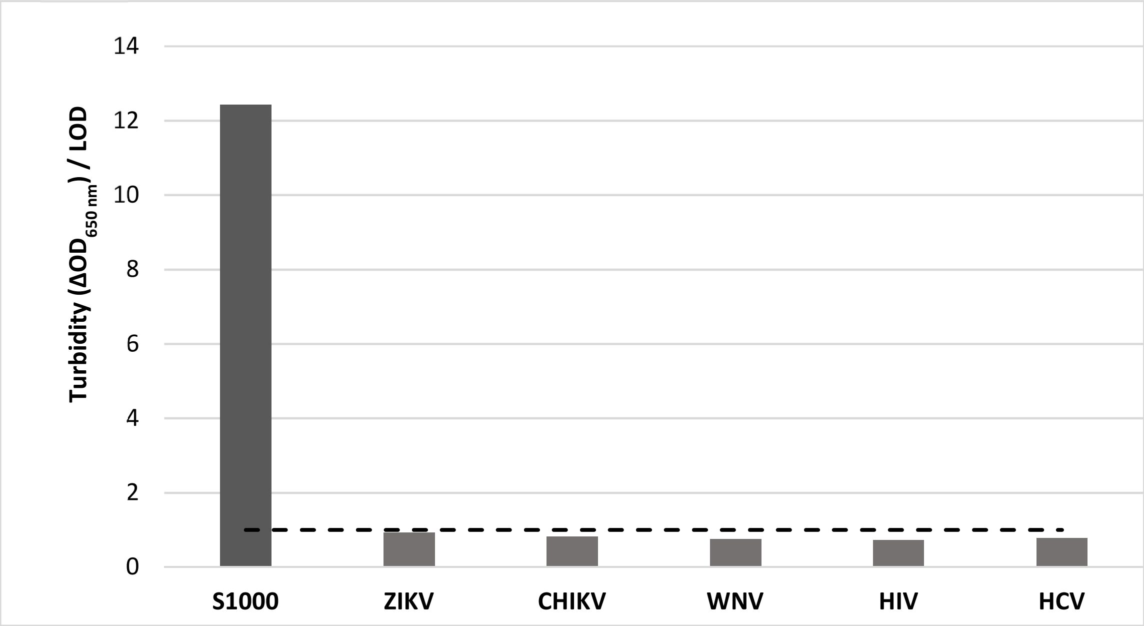

Supplement: Supplementary file 1 [file microorganisms-09-00674-s001.pdf]
